# Emergency tracheal intubation during off-hours is not associated with increased mortality in hospitalized patients: a retrospective cohort study

**DOI:** 10.1186/s12871-020-01188-3

**Published:** 2020-10-21

**Authors:** Jun-Le Liu, Jian-Wen Jin, Zhong-Meng Lai, Jie-Bo Wang, Jian-Sheng Su, Guo-Hua Wu, Wen-Hua Chen, Liang-Cheng Zhang

**Affiliations:** 1 Department of anesthesiology, Union Hospital, Fujian Medical University, XinQuan Road 29th, Fuzhou, 350001 Fujian China; 2 Department of Clinical Medicine, Fujian Health College, 366th GuanKou, Fuzhou, 350101 Fujian China

**Keywords:** Emergent endotracheal intubation, Mortality, Off-hours

## Abstract

**Background:**

The prognosis of hospitalized patients after emergent endotracheal intubation (ETI) remains poor. Our aim was to evaluate the 30-d hospitalization mortality of subjects undergoing ETI during daytime or off-hours and to analyze the possible risk factors affecting mortality.

**Methods:**

A single-center retrospective study was performed at a university teaching facility from January 2015 to December 2018. All adult inpatients who received ETI in the general ward were included. Information on patient demographics, vital signs, ICU (Intensive care unit) admission, intubation time (daytime or off-hours), the department in which ETI was performed (surgical ward or medical ward), intubation reasons, and 30-d hospitalization mortality after ETI were obtained from a database.

**Results:**

Over a four-year period, 558 subjects were analyzed. There were more male than female in both groups (115 [70.1%] vs 275 [69.8%]; *P* = 0.939). A total of 394 (70.6%) patients received ETI during off-hours. The patients who received ETI during the daytime were older than those who received ETI during off-hours (64.95 ± 17.54 vs 61.55 ± 17.49; *P* = 0.037). The BMI of patients who received ETI during the daytime was also higher than that of patients who received ETI during off-hours (23.08 ± 3.38 vs 21.97 ± 3.25; *P* < 0.001). The 30-d mortality after ETI was 66.8% (373), which included 68.0% (268) during off-hours and 64.0% (105) during the daytime (*P* = 0.361). Multivariate Cox regression analysis found that the significant factors for the risk of death within 30 days included ICU admission (HR 0.312, 0.176–0.554) and the department in which ETI was performed (HR 0.401, 0.247–0.653).

**Conclusions:**

The 30-d hospitalization mortality after ETI was 66.8%, and off-hours presentation was not significantly associated with mortality. ICU admission and ETI performed in the surgical ward were significant factors for decreasing the risk of death within 30 days.

**Trial registration:**

This trial was retrospectively registered with the registration number of ChiCTR2000038549.

## Background

Emergent endotracheal intubation (ETI) for most hospitalized patients with critical illnesses is often performed to stabilize patients’ vital signs. Despite the potentially beneficial effects of ETI, such as better control of ventilation and oxygenation as well as protection from aspiration, the outcomes after ETI remain poor [1]. Along with for the primary disease of the patient, some factors may affect prognosis, such as performing endotracheal intubation at the opportune moment and location, performance by a sophisticated anesthesiologist, and emergency treatments after ETI.

A previous study indicated that admission during the weekend was associated with a significantly increased mortality compared with midweek admission [2–4]. A shortage of medical staff may be a serious problem on the weekend. At most medical institutions, including our own, staffing levels dramatically decrease during off-hours. At these times, staff performance may be impaired because of fatigue and disrupted circadian rhythms [5]. Furthermore, physicians who work during off-hours also provide coverage to patients with whom they may be less familiar. The impact of shift work, particularly during the nighttime, has been shown to impact psychomotor skills and the performance of skilled activities, such as cardiopulmonary resuscitation [5, 6]. However, using a national database in Japan, Jneid et al. found no significant differences between patients with acute myocardial infarction who presented during regular or off-hours [7]. Furthermore, the causes of worse outcomes during off-hours in real-world settings remain uncertain. Presumably, a difference in human and technical resources during different times is possible, and the problem might not only be that there are fewer trained health providers but also that professionals are tired and that there are other factors influencing prognosis [8].

To date, studies on the association between off-hours presentation and ETI-related outcomes have been limited, to the best of our knowledge, and we sought to clarify the association between inpatients undergoing ETI during off-hours and mortality.

The primary goal of this study was the 30 days mortality of inpatients after ETI during the daytime or off-hours; the secondary goal was to analyze the risk factors affecting mortality.

## Methods

### Study setting and design

This single-center retrospective cohort study was undertaken to explore the outcomes of inpatients following ETI from January 2015 to December 2018 in the general ward of the Union Hospital, Fujian Medical University, China. (ChiCTR2000038549) The hospital has 2500 beds and serves as a university teaching facility. This study was conducted in accordance with the amended Declaration of Helsinki. Before data collection, the Research Ethics Committee of the hospital approved this study and waived the requirement for informed consent.

All hospitalized patients (aged ≥18 years) who underwent ETI in the general ward were included. Patients were excluded if they were intubated prior to admission, had preexisting endotracheal tube exchanges, were less than 18 years old, were intubated in the ICU or emergency department, had incomplete data, etc.

### Operation procedure of emergency endotracheal intubation

Our special endotracheal intubation rescue team consisting of an experienced attending anesthesiologist and an anesthesia intern were the first responders for all emergent airway requests in our hospital. In addition to the team on call, a variety of video laryngoscope must been equipped. All patients were intubated by video laryngoscope under emergency circumstances.

### Clinical data collection

Demographic data were extracted from the medical record, including age, sex, body mass index (BMI), and admission diagnosis. Factors related to intubation included the preintubation heart rate (HR), mean arterial pressure (MAP), oxygen saturation (SPO_2_), shock index (SI), ICU admission, preintubation cardiopulmonary cerebral resuscitation (CPCR), postintubation CPCR, intubation time (the daytime was defined as between 8:00 AM and 6:00 PM from Monday to Friday; off-hours was defined as the period from 6:01 PM to 7:59 AM from Monday through Friday plus the entire weekend), and intubation reasons. We also recorded the 1-d, 7-d, 30-d mortality after ETI and the reasons for mortality.

Data were extracted into a standardized data form by 3 separate reviewers (JB Wang, JS Su, and GH Wu) who were blinded to the study hypotheses.

### Outcome measures

The primary goal of this study was to evaluate the 30-d mortality of inpatients after ETI during the daytime or off-hours; the secondary goal was to analyze the risk factors affecting mortality. The factors included age (≥65 years = 0;18–64 years = 1), sex (female = 0; male = 1), BMI (18.5–23.9 = 0; <18.5 or ≥ 24 = 1), time of ETI (daytime = 0; off-hours = 1), department of ETI (surgery ward = 0; medicine ward = 1), characteristics Pre-ETI [CPCR (no = 0; yes = 1), Consciousness (no = 0; yes = 1), MAP(≥70 mmHg = 0; <70 mmHg = 1), HR(≥60 or<100 bate/min = 0; <60 or ≥ 100 bate/min = 1), and SI(≤1 or >2 = 0; 1–2 = 1)], CPCR (no = 0; yes = 1), and ICU admission (no = 0; yes = 1) post-ETI.

### Statistical analysis

For continuous parameters, Student’s t-tests were used to evaluate differences between groups; for discontinuous parameters, chi-square statistics were used to detect differences between groups.

The secondary outcomes were evaluated with the log-rank test and cox regression model of survival analysis. The significance level was set at 5% (*P* < 0.05) for all statistical tests. Data were coded and stored in Excel and were analyzed using the Statistical Package for the Social Sciences version 21.0 software (SPSS Inc., Chicago, IL, USA). All results are presented as numbers (median and percentage), ratios or the mean ± the standard deviation, unless otherwise noted.

## Results

### Demographics and patient characteristics

**Fig. 1:**
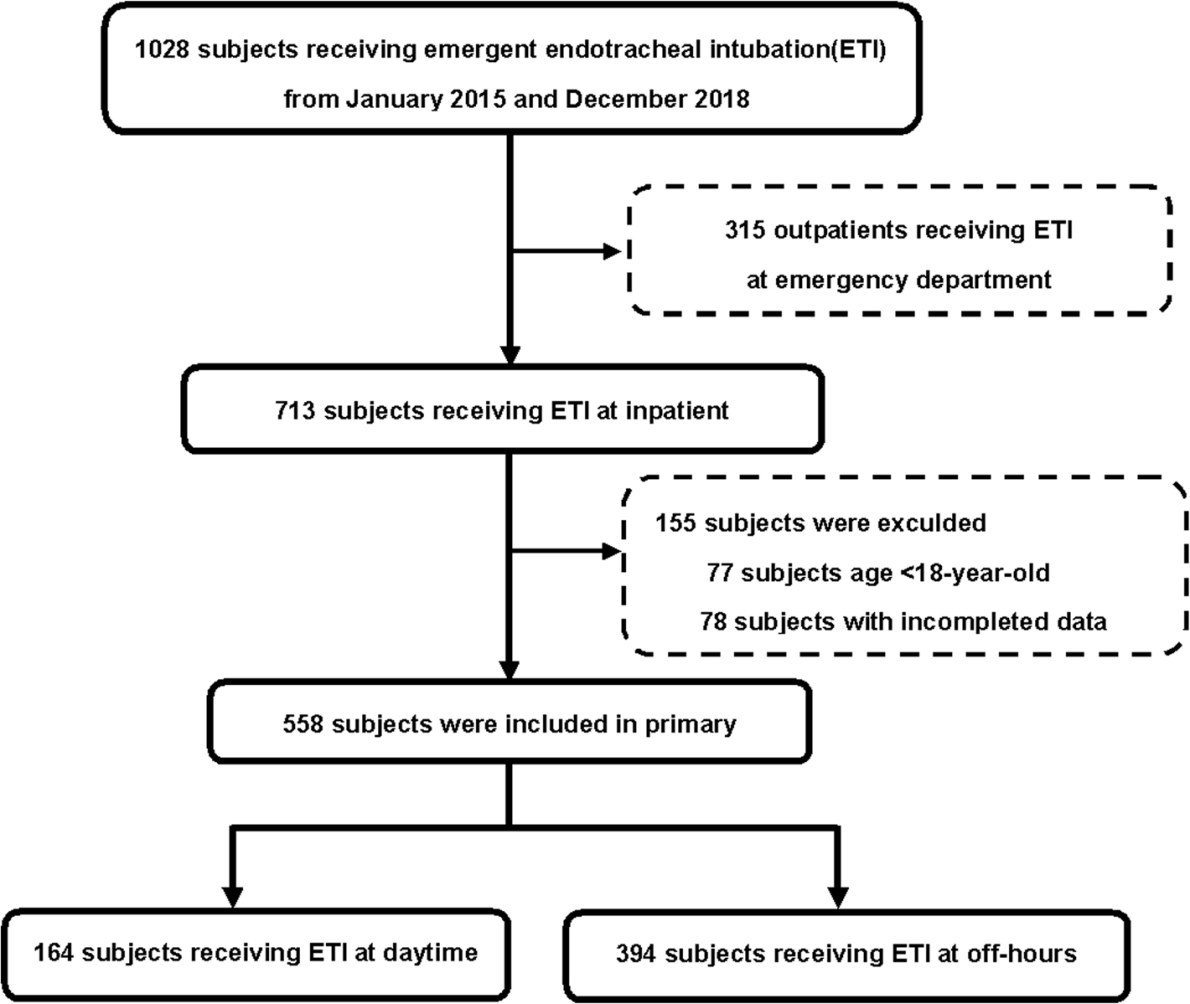
Flowchart showing subjects enrollment and analysis

Over a four-year period, there were 1028 subjects who underwent emergent endotracheal intubation. Among these patients, we excluded 470 subjects for the following reasons: intubation in the emergency department (315), age less than 18 years (77), and incomplete data (78). The remaining 558 subjects were analyzed (Fig. 1).

Table 1 shows the clinical demographics of the 558 subjects who underwent emergent endotracheal intubation. A total of 394 (70.6%) patients received ETI during off-hours. There were more male than female in both groups (115 [70.1%] vs 275 [69.8%]; *P* = 0.939). The patients who received ETI during the daytime were older than those who received ETI during the off-hours (64.95 ± 17.54 vs 61.55 ± 17.49; *P* = 0.037). The BMI of the patients who received ETI during the daytime was also higher than that of the patients who received ETI during off-hours (23.08 ± 3.38 vs. 21.97 ± 3.25; *P* < 0.001). The most frequent admitting diagnosis was neurological diseases, followed by heart diseases, gastrointestinal diseases, and end-stage hematopathy. There were no differences in the admitting diagnoses of the subjects during the daytime or off-hours.

**Table 1:** Demographic data of inpatients who underwent emergent endotracheal intubation

Characteristics	Daytime (*N* = 164)	Off-hours (*N* = 394)	*P*
Male Gender^a^	115 (70.1%)	275 (69.8%)	0.939
Age (median, years)^b^	67 (22–96)	64 (20–96)	0.037
BMI (kg/m^2^)^b^	23.08 ± 3.38	21.97 ± 3.25	0.000
Admitting diagnosis^a^			
Heart disease	34 (20.7%)	64 (16.2%)	0.204
CAD	25 (15.2%)	54 (13.7%)	0.635
AMI	13 (7.9%)	33 (8.4%)	0.861
Neurological diseases	48 (29.3%)	96 (24.4%)	0.228
cerebral hemorrhage	5 (3.0%)	23 (5.8%)	0.169
cerebral infarction	17 (10.4%)	24 (6.1%)	0.078
End stage Hematopathy	16 (9.8%)	59 (15.0%)	0.100
Respiratory diseases	17 (10.4%)	43 (10.9%)	0.849
COPD	4 (2.4%)	17 (4.3%)	0.289
pneumonia	6 (3.7%)	13 (3.3%)	0.831
Gastrointestinal diseases	27 (16.5%)	67 (17.0%)	0.876
obstruction	3 (1.8%)	12 (3.0%)	0.602
perforation	2 (1.2%)	1 (0.3%)	0.208
Burn	6 (3.7%)	9 (2.3%)	0.361
Orthopaedics	3 (1.8%)	9 (2.3%)	0.986
Urology	4 (2.4%)	19 (4.8%)	0.291
CTD	2 (1.2%)	7 (1.8%)	0.915
Others	7 (4.3%)	21 (5.3%)	0.601

Data are presented as numbers (median and percentage), or ratios or mean ± standard deviation

*BMI* Body mass index, *ACD* Coronary artery disease, *AMI* Acute myocardial infarction, *COPD* Chronic obstructive pulmonary disease, *ARDS* Acute respiratory distress syndrome, *CTD* Connective tissue disease

^a^Chi-square

^b^two-tailed Student’s t test

Table 2 shows the intubating factors and characteristics of the hospitalized patients who underwent emergent endotracheal intubation. There was no difference between the patients who underwent ETI during the daytime and off-hours who were admitted to the ICU [74/164 (45.1%) vs 157/394 (39.8%), *P* = 0.249]. Of the patients who received ETI in the surgical ward, 42 (25.6%) patients received ETI during the daytime, and 127 (32.2%) patients received ETI during off-hours (χ^2^ = 2.406; *P* = 0.121). The most common causes of ETI were respiratory diseases, followed by cardiovascular diseases and neurological diseases. There were no differences in the causes of ETI between the daytime and off-hours. There were 149 (26.7%) inpatients who underwent cardiopulmonary cerebral resuscitation (CPCR) before ETI (51 [31.1%] vs 98 [24.9%], *P* = 0.130), and 167 (29.9%) inpatients underwent CPCR after ETI (51 [31.1%] *vs*116 [29.4%], *P* = 0.697). Pre-ETI characteristics, such as HR, MAP, SPO_2_, and SI, were not different between the two groups.

**Table 2:** Intubating characteristics of inpatients who underwent emergent endotracheal intubation

Characteristics	Daytime (*N* = 164)	Off-hours (*N* = 394)	*X*^*2*^ *or* 95% CI	*P*
ICU admission	74 (45.1%)	157 (39.8%)	1.328	0.249
Departments of ETI			2.406	0.121
Surgical ward	42 (25.6%)	127 (32.2%)		
Medical ward	122 (74.4%)	267 (67.8%)		
Mortality after ETI				
1 day	65 (39.6%)	164 (41.6%)	0.190	0.663
7 days	85 (51.8%)	218 (55.3%)	0.572	0.450
30 days	105 (64.0%)	268 (68.0%)	0.834	0.361
Reasons for intubation				
Respiratory diseases	90 (54.9%)	239 (60.7%)	1.600	0.206
Cardiovascular diseases	60 (36.6%)	123 (31.2%)	1.514	0.219
Neurological diseases	14 (8.5%)	26 (6.6%)	0.653	0.420
Others	1 (0.6%)	5 (1.3%)	0.056	0.812
CPCR				
Pre-ETI	51 (31.1%)	98 (24.9%)	0.292	0.130
Post-ETI	51 (31.1%)	116 (29.4%)	0.151	0.697
Pre-ETI characteristics				
HR (BPM)	116.051 ± 35.019	114.635 ± 32.443	−4.654 to 7.482	0.647
MAP (mmHg)	83.555 ± 27.597	85.071 ± 26.202	−6.370 to 3.344	0.540
SPO_2_ (%)	79.701 ± 16.496	79.558 ± 16.459	−2.868 to 3.152	0.927
SI	0.993 ± 0.424	0.984 ± 0.413	−0.064 to 0.093	0.736

Data are presented as numbers (median and percentage), or ratio or mean ± standard deviation

*ICU* Intensive care unit, *ETI* Emergent endotracheal intubation, *CPCR* Cardiopulmonary cerebral resuscitation, *HR* Heart rate, *MAP* Mean arterial pressure, *SPO*_*2*_ Pulse oxygen saturation, *SI* Shock index

^a^Chi-square

^b^two-tailed Student’s *t* test

### Mortality

**Fig. 2:**
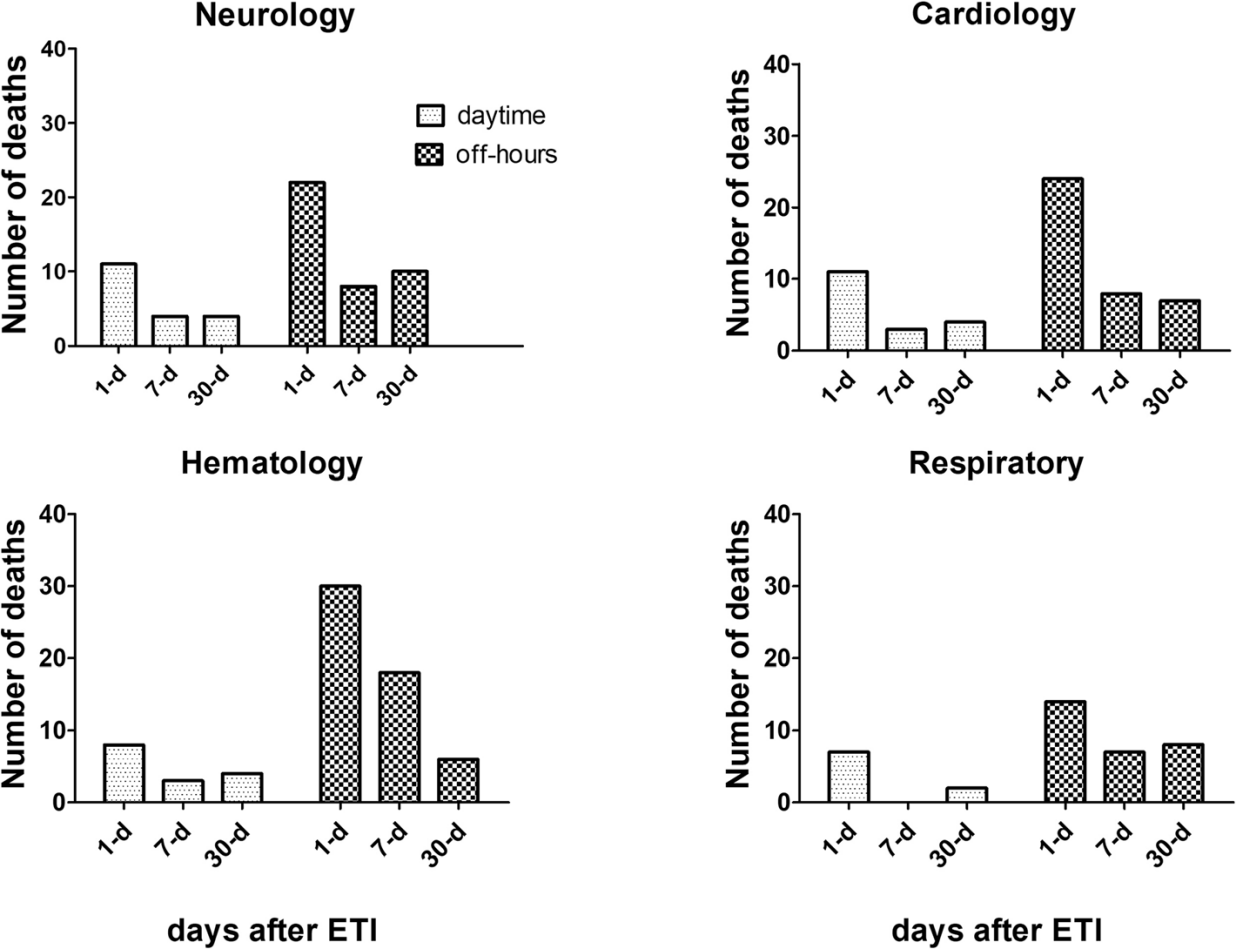
Number of deaths after ETI over time between daytime group and off-hours group among Neurology, Cardiology, Hematology, and Respiratory departments

Overall, the 1-day, 7-day and 30-d mortality after ETI were 41.0% (229), 54.3% (303), and 66.8% (373), respectively. The departments with the most ETI-related deaths were the Neurology, Cardiology, Hematology, and Respiratory departments. Detailed data distributions are shown in Fig. 2.

### One-day mortality

The 1-d mortality was 39.6% (65/164) during the daytime and 41.6% (164/394) during off-hours (χ^2^ = 0.190, *P* = 0.663). The most common causes of mortality were cardiopulmonary arrest (40.0%, 26/65), respiratory failure (23.1%, 15/65), and heart failure (9.2%, 6/65) during the daytime, which is in accordance with cardiopulmonary arrest (40.9%, 67/164), respiratory failure (20.7%, 34/164), and heart failure (12.2%, 29/164) during off-hours. There were no significant differences in the causes of death between the two groups (Table 3).

**Table 3:** Causes of death in hospitalization patients who underwent emergent endotracheal intubation

	Daytime (*N* = 105)	Off-hours (*N* = 268)	*X*^*2*^	*P*
***1-day after ETI***	65 (61.9%)	164 (61.2%)	0.016	0.899
Respiratory failure	15 (23.1%)	34 (20.7%)	0.152	0.696
CA	26 (40.0%)	67 (40.9%)	0.014	0.906
Heart failure	6 (9.2%)	20 (12.2%)	0.406	0.524
AMI	3 (4.6%)	5 (3.0%)	0.034	0.855
MODS	4 (6.2%)	13 (7.9%)	0.033	0.856
Septic shock	4 (6.2%)	9 (5.5%)	0.015	0.904
Brain failure	3 (4.6%)	11 (6.7%)	0.085	0.772
PE	3 (4.6%)	1 (0.6%)	2.330	0.127
DIC	1 (1.5%)	4 (2.4%)	0.007	0.935
***7-day after ETI***	20 (19.0%)	54 (20.1%)	0.058	0.810
Respiratory failure	9 (45.0%)	26 (48.1%)	0.058	0.810
CA	4 (20.0%)	3 (5.6%)	2.069	0.150
Heart failure	2 (10.0%)	5 (9.3%)	0.123	0.726
AMI	0 (0%)	2 (3.7%)		1.000
MODS	2 (10.0%)	8 (14.8%)	0.024	0.877
Septic shock	2 (10.0%)	6 (11.1%)	0.081	0.774
Brain failure	1 (5.0%)	4 (7.4%)	0.024	0.877
***30-day after ETI***	20 (19.0%)	50 (18.7%)	0.008	0.931
Respiratory failure	7 (35.0%)	14 (28.0%)	0.333	0.564
CA	1 (5.0%)	4 (8.0%)	0.005	0.942
Heart failure	4 (20.0%)	13 (26.0%)	0.049	0.826
AMI	1 (5.0%)	2 (4.0%)		0.642
MODS	5 (25.0%)	4 (8.0%)	2.324	0.127
Septic shock	2 (10.0%)	7 (14.0%)	0.003	0.955
Brain failure	0 (0%)	6 (12.0%)	1.317	0.251

Data are presented as number (percentage) or ratio

*ETI* Emergent endotracheal intubation, *CA* Cardiopulmonary arrest, *AMI* Acute myocardial infarction, *MODS* Multiple organ dysfunction syndrome, *PE* Pulmonary embolism, *DIC* Diffuse intravascular coagulation. Chi-square test

### Seven-day mortality

The 7-d mortality was 51.8% (85/164) during the daytime and 55.3% (218/394) during off-hours (χ^2^ = 0.572, *P* = 0.450). The number of deaths within 7 days after ETI was 20/105 (19.0%) during the daytime and 54/268 (20.1%) during off-hours (χ^2^ = 0.058, *P* = 0.810). The most common causes were respiratory failure (45.0%, 9/20) and cardiopulmonary arrest (20.0%, 9/20) during the daytime, whereas the most common causes were respiratory failure (48.1%, 26/54), MODS (14.8%, 8/54) and septic shock (11.1%, 6/54) during off-hours. There were no significant differences in the causes of death within 7 days after ETI between the two groups. Among the deaths within 7 days after ETI during the daytime or off-hours, the department distribution is shown in Fig. 2.

### Thirty-day mortality

The 30-d mortality between the two groups was not significantly different (64.0% [105/164] vs 68.0% [268/394]; χ^2^ = 0.834, *P* = 0.361). The number deaths within 7–30 days after ETI was 20/105 (19.0%) during the daytime and 50/268 (18.7%) during off-hours (χ^2^ = 0.008, *P* = 0.931). The most common causes were respiratory failure (35.0%, 7/20), MODS (25.0%, 5/20), and heart failure (20.0%, 4/20) during the daytime, whereas the most common causes were respiratory failure (28.0%, 14/50), heart failure (26.0%, 13/50), and septic shock (14.0%, 7/50) during off-hours. There were no significant differences in the causes of death within 30 days after ETI between the two groups.

### Some risk factors for mortality

**Fig. 3:**
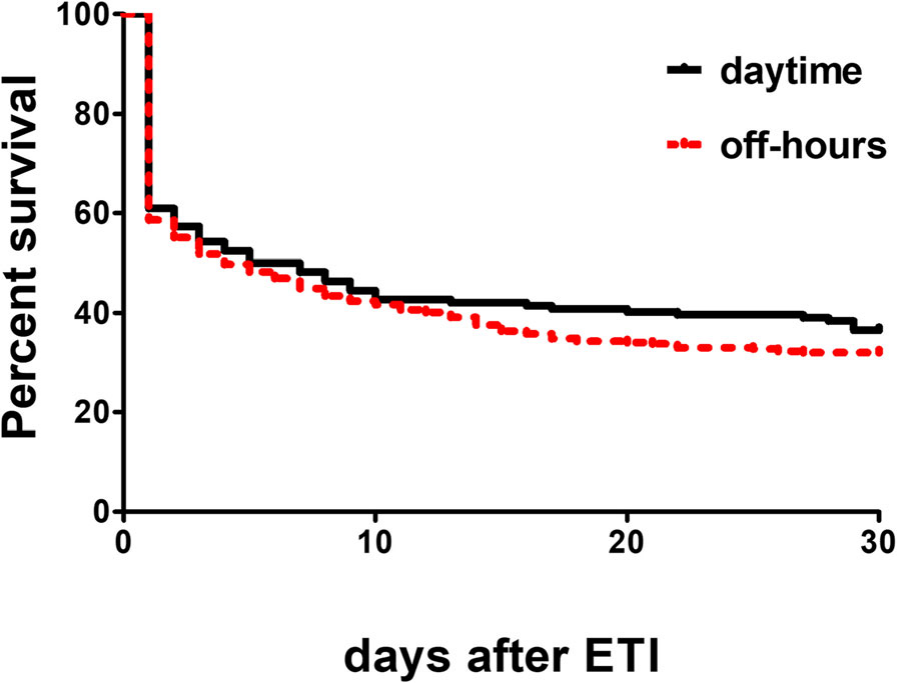
The Kaplan-Meier survival curves of the cumulative probability of death within 30 days after ETI between daytime group and off-hours group

Figure 3 shows the Kaplan-Meier survival curves of the cumulative probability of death within 30 days after emergent endotracheal intubation during the daytime or off-hours (hazard ratio 0.879, 0.679–1.137). Table 4 shows the results of the multivariate Cox regression analysis. A significant factor for a decreased risk of death at 30 days was ICU admission—patients who were admitted to the ICU were at a decreased risk compared with those who were not admitted to the ICU (hazard ratio 0.312, 0.176 to 0.554). The departments in which ETI was performed (medical wards or surgical wards) were also a significant factor that affected the risk of death at 30 days (hazard ratio 0.401, 0.247 to 0.653).

**Table 4:** Cox regression model of survival analysis of variables for 30-day mortality

	Hazard ratio	95.0% CI	Sig.
Age (years)	1.096	0.656–1.830	0.727
Sex	1.491	0.877–2.535	0.140
BMI (kg/m^2^)	1.003	0.598–1.683	0.991
Time of ETI	1.327	0.730–2.413	0.354
Departments of ETI	0.401	0.247–0.653	0.000
Pre-ETI			
CPCR	1.389	0.316–6.101	0.663
Consciousness	1.090	0.655–1.813	0.739
MAP (mmHg)	2.761	0.867–8.789	0.086
HR (mmHg)	1.043	0.548–1.986	0.898
SI	1.214	0.701–2.105	0.489
Post-ETI			
CPCR	1.849	0.595–5.748	0.288
ICU	0.312	0.176–0.554	0.000

*Abbreviation*: *CI* Confidence interval, *Sig* significance, *BMI* Body mass index, *ETI* Emergent endotracheal intubation, *CPCR* Cardiopulmonary cerebral resuscitation, *MAP* Mean arterial pressure, *HR* Heart rate, *SI* Shock index, *ICU* Intensive care unit

## Discussion

The major findings of this study were as follows. First, the 30-d hospitalization mortality after ETI was as high as 66.8%, and off-hours presentation was not significantly associated with mortality. Second, ICU admission and ETI performed in the surgical ward were significant factors for decreasing the risk of death within 30 days.

In our study, the median age of patients who received ETI during the daytime was higher than that during off-hours. Advanced age is typically positively associated with a worse prognosis. Nevertheless, some studies found that patients during off-hours may be characterized by a higher severity than their counterparts during daytime due to comorbidities and complications [9]. This difference in mortality cannot be ruled out as being a result of age and complicated diseases. The multivariate Cox regression analysis did not show that age was a significant factor for the risk of death at 30 days.

The BMI of patients who received ETI during the daytime was much higher than that of patients who received ETI during off-hours in our study. However, there was no consistent trend in mortality. Previous papers indicated that a higher BMI may be associated with the number of difficult airways [10, 11] . Unfortunately, we did not analyze the number of difficult airway cases in this study. There were three patients with difficult endotracheal intubations over a four-year period. One patient with severe ankylosing spondylitis needed to receive ETI because of severe pulmonary infections and respiratory failure. Another two patients had severe head and face burns. With consciousness and autonomous breathing, these patients were successfully given endotracheal intubation by fiberoptic bronchoscope. Kim et al. suggested that intervention by a medical emergency team could reduce emergent endotracheal intubation complications, such as hypotension, esophageal intubation, and the aspiration of gastric contents, from 41.7 to 18.1% in the general ward [12] . With fiber laryngoscopy, an emergency team consisting of an experienced attending anesthesiologist and an anesthesia intern were the first responders for all emergent airway requests at our hospital.

The most common causes of ETI were respiratory diseases, followed by cardiovascular diseases and neurological diseases in our study, which was in accordance with a previous survey [13–15] . However, the most common admitting diagnosis of the subjects was neurological diseases, heart diseases, gastrointestinal diseases, and end-staged hematopathy. One important factor was that neurology, cardiology, and hematology are our largest departments, and there are over 500 beds in these departments.

Our study indicated that the 30-d mortality after ETI was 66.8%, and the 1-d mortality accounted for 61.4% of the total 30-d mortality. Gabriel Wardi et al. demonstrated that the hospitalization mortality was 40.7% for patients undergoing ETI, which was far lower than the levels in our study [16] .There are several factors that may explain our high mortality. First, health care is still at a low level in China, and a shortage of medical staff has persisted for a long time. Second, many subjects with comorbidities and complications were in critical decompensated conditions before admission. Many of these patients were end-stage, and no treatments could have changed their poor outcomes. Furthermore, in China, the customs and folk traditions do not allow the dead to enter their village, so their families asked for the medical staff to give the dying patients emergent endotracheal intubation before being discharged.

We did not find significant differences in the mortality of patients who underwent ETI in the daytime and off-hours. A previous study indicated that admission on the weekend was associated with a significantly increased mortality compared with that of a midweek admission [2, 17, 18] . Various factors have been attributed to the difference between mortality in daytime and off-hours, such as the availability of senior specialists, the number of skilled nursing staff, and human factors, such as sleep deprivation and fatigue [19, 20] . The different quality and system of medical services might be associated with poor prognosis. Whether physicians and surgeons share common decision-making characteristics remains unclear. Sometimes, experiential decision making is a necessity for rapid, life-saving decision making (e.g., to defibrillate or perform CPCR), and other scenarios may change how to address some complicated management issues (e.g., the decision to admit to the ICU or perform ETI on patients with multiple comorbidities) [21]. In our study, more patients in the surgical ward after ETI were admitted to the ICU, and the SPO_2_ before ETI was also obviously higher in the surgical ward than in the medical ward. We cannot rule out the effect of clinical decision making on mortality.

The cox regression model of survival analysis showed that ICU admission was a significant factor for decreasing the risk of death within 30 days. Survival rates are gradually increasing, and the prognosis has improved due to highly qualified personnel and technology in the ICU, where critical patients are followed up [22]. Artificial respiration support is provided through mechanical ventilators in addition to many life-saving medical procedures, such as peritoneal dialysis/hemodialysis, plasmapheresis, extracorporeal membrane oxygenation and various surgical operations. Jaber et al recently reported that the presence of backup staff was independently associated with a reduced risk of complications related to ETI performed in the intensive care unit [23].

ETI performed in the surgical ward was also a significant factor for decreasing the risk of death within 30 days based on cox regression model of survival analysis. In our study, 7-d and 30-d mortalities after ETI in the surgical ward were much lower than those in the medical ward. In addition to a larger proportion of surgical patients who were admitted to the ICU and received more sophisticated management, there may be some other factors. For example, the conditions of surgical patients are always dangerous, but hypoxia or unstable blood flow may be a consequence of surgery and other related factors. As soon as the reasons for surgery are resolved, cardiopulmonary function gradually improves to a normal state. Bergum et al. demonstrated that survival significantly increased if hypoxia is appropriately treated [24]. However, the conditions of some medical patients are always fragile, deteriorating or end-stage. Even with mechanical ventilation and cardiopulmonary cerebral resuscitation, it is rarely possible to immediately relieve conditions of hypoxia and hemodynamic instability.

We excluded some patients who received ETI in the emergency department and outside of the hospital. In contrast to the typical in-hospital setting, the scenario of endotracheal intubation performed in outside of the hospital environment or in the emergency department is usually fraught with unique challenges, such as vomit-flooded airways without adequate suctioning equipment, ground-level patient positions, or confined spaces. Furthermore, data acquisition for these patients may be incomplete and inaccurate.

There are several limitations to our study. First, this retrospective study was conducted in a single center, and the results of the current analysis might not be generalizable to other hospitals with different medical staff and patient populations. Second, the time from the receipt of the intubation request to the completion of intubation is critical to the prognosis of patients. Unfortunately, our retrospective analysis did not record these data accurately. The absence of some information and incomplete data made it impossible to analyze this relationship. Last, we estimated only the 30-d hospitalization mortality of patients who received ETI and roughly analyzed the possible causes of mortality and the correlation to the daytime and off-hours where ETI was performed. Some of these factors are not consequences. Further studies must be performed to clarify the key factors affecting mortality, and some necessary measures must be performed to reduce the mortality of these critical patients.

## Conclusions

The 30-d hospitalization mortality after ETI was 66.8%, and off-hours presentation was not significantly associated with mortality. ICU admission and ETI performed in the surgical ward were significant factors for decreasing the risk of death within 30 days.

## Data Availability

The datasets used and/or analysed during the current study available from the corresponding author on reasonable request.

## Abbreviations

ETI: Emergent endotracheal intubation
BMI: Body mass index
ACD: Coronary artery disease
AMI: Acute myocardial infarction
COPD: Chronic obstructive pulmonary disease
ARDS: Acute respiratory distress syndrome
CTD: Connective tissue disease
ICU: Intensive care unit
CPCR: Cardiopulmonary cerebral resuscitation
HR: Heart rate
MAP: Mean arterial pressure
SPO2: Pulse oxygen saturation
SI: Shock index
CA: Cardiopulmonary arrest
MODS: Multiple organ dysfunction syndrome
PE: Pulmonary embolism
DIC: Diffuse intravascular coagulation
CI: Confidence interval
Sig: significance
